# Blockchain-Based Supply Chain Systems, Interoperability Model in a Pharmaceutical Case Study

**DOI:** 10.3390/s23041962

**Published:** 2023-02-09

**Authors:** Yeray Mezquita, Blaž Podgorelec, Ana Belén Gil-González, Juan Manuel Corchado

**Affiliations:** 1 BISITE Digital Innovation Hub, Department of Informatics and Automatics, University of Salamanca, Edificio Multiusos I+D+I, 37007 Salamanca, Spain; 2 Institute of Applied Information Processing and Communications, Graz University of Technology, 8010 Graz, Austria

**Keywords:** blockchain, verifiable credentials, supply chain, traceability, interoperability

## Abstract

The main purpose of supply chain systems based on blockchain technology is to take advantage of technology innovations to ensure that a tracked asset’s audit trail is immutable. However, the challenge lies in tracking the asset among different blockchain-based supply chain systems. The model proposed in this paper has been designed to overcome the identified challenges. Specifically, the proposed model enables: (1) the asset to be tracked among different blockchain-based supply-chain systems; (2) the tracked asset’s supply chain to be cryptographically verified; (3) a tracked asset to be defined in a standardized format; and (4) a tracked asset to be described with several different standardized formats. Thus, the model provides a great advantage in terms of interoperability between different blockchain-driven supply chains over other models in the literature, which will need to replicate the information in each blockchain platform they operate with, while giving flexibility to the platforms that make use of it and maintain the scalability of those logistic platforms. This work aims to examine the application of the proposed model from an operational point of view, in a scenario within the pharmaceutical sector.

## 1. Introduction

Blockchain technology is being used for the development of traceability systems in supply chains. Features such as immutability, integrity, and transparency ensure that the information used in the supply chain process is authentic and reliable. According to studies by [1,2,3,4], blockchain technology contributes to more sustainable and transparent management in supply chains.

In [5], the authors emphasize the five main benefits of traceability systems: informational security, technological advantages, improvement of collaboration and trust, reduced economic loss and product waste, and improved sustainability and transparency. The study by [6] also demonstrated that projects and initiatives are already capable of establishing transparent and more sustainable asset production and distribution through the use of blockchain technology. Furthermore, the utilization of blockchain technology in the supply chain is becoming more frequent, and the technology appears to be promising in achieving transparency in supply chains [4,7].

It was found in [5] that current blockchain-based supply chain solutions lack standardization and flexibility, i.e., the possibility that the tracked asset-related data are defined and written in a standardized form (e.g., GS1 (https://www.gs1.org/, accessed on 10 January 2023)). Additionally, a survey by [8] discusses the fact that interoperability and the lack of data in a standardized format and storage problems pose challenges to blockchain-based supply chains. Moreover, another explanatory study by [9] reached a similar conclusion: achieving interoperability in blockchain-driven supply chains is a challenge that must be addressed.

The objective of this manuscript is to propose a novel model that offers a solution to the interoperability and standardization challenges of blockchain-based supply chain systems. The use of blockchain technology in pharmaceutical and medical equipment supply chains is becoming more popular [10], but also entails certain risks. In [11], the authors divide the risks of this technology into three groups: organizational, social, and technological threats. Focusing on the framework we have proposed, Section 4 addresses the organizational issues group—specifically, the problems caused by the lack of interoperability between different blockchain-based solutions. We propose a use case in a pharmaceutical scenario, not only because it is a popular topic in the scientific community, but also because it is a well-known scenario to the authors. The upside of the proposed model is that the findings could be extrapolated to any other logistics-based use case scenario.

Thanks to the proposed model, every asset tracked within a blockchain-based supply chain system is represented as a verifiable credential. The model is adapted for usage in supply chain systems and follows the recommendation for expressing verifiable credentials on the Web published by World Wide Web Consortium. Except for the system performance challenges related to the storage capacity problem, which have been addressed within the model’s design, other areas are beyond the scope of this article.

The research objective of our paper is to find a way to improve the interoperability of blockchain-based supply chain platforms found in the literature. The contribution of this paper can be summarized as the creation of a novel model that:

- Enables interoperability among different blockchain-based supply chain systems;
- Enables the data related to every asset that is tracked within a blockchain-based supply chain to be defined in different standardized formats;
- Stores all the data related to the asset tracked in a blockchain-based supply chain in an off-chain manner.

The rest of the paper is structured as follows: Section 2 describes previous studies that addressed the challenge of asset interoperability within one or more logistics systems. The proposed model is described in Section 3. The experimentation carried out with the proposed model is outlined in Section 4. There, the workflow of the system is described in more detail with a hypothetical use case scenario of the proposed framework in which defined actors use different blockchain technologies for the tracking of their assets. In Section 5, the results of the conducted experiment are discussed, along with the answers found to the posed hypotheses. Finally, conclusions are drawn from the research conducted in Section 6.

## 2. Literature Study

The adoption of blockchain technology by the logistics sector is leading to the emergence of different environments and platforms, whose stakeholders maintain a blockchain network for the traceability of their assets. However, it is not possible to exchange assets between two blockchain systems that have different information storage methods. State-of-the-art research points to the need to develop and use standards for supply chain systems to enable interoperability between blockchain solutions [12].

In this section, we describe the methodology used to identify published research that is related to the topic of this paper, namely, to interoperability in blockchain-based supply chain systems. The following two research questions were considered to assess the interoperability of different blockchain-based supply chain systems and tracked asset  specification:

RQ 1. Is it possible to achieve interoperability between different blockchain-based supply chain systems?
RQ 2. Is it possible to specify tracked assets with different standardized formats?

Considering the research questions, the following four hypotheses have been defined:

**Hypothesis 1.**  *With the proposed model, the assets can be tracked across different blockchain-based supply chain systems. This hypothesis directly maps onto RQ1 and is vital to the interoperability capability between blockchain-based supply chains.*

**Hypothesis 2.**  *With the proposed model, the supply chain of the tracked asset can be cryptographically verified despite different blockchain networks being involved. This hypothesis directly maps onto RQ1 and relates to the ability of the blockchain-based platforms to allow the tracking of the assets.*

**Hypothesis 3.**  *With the proposed model, tracked assets can be defined in standardized formats. This hypothesis directly maps onto RQ2 and relates to the capability of the proposed model to define the assets in any kind of standardized format.*

**Hypothesis 4.**  *With the proposed model, the same asset tracked among different blockchain-based supply chain systems can be defined with many different standardized formats. This hypothesis directly maps onto RQ2 and relates to the freedom that the model offers to choose different standard formats even for the same tracked asset.*

To verify the hypotheses and justify the novelty of the proposed model, we conducted a literature review where we searched in ScienceDirect (https://www.sciencedirect.com/, accessed on 6 November 2022), Springer-link (https://link.springer.com/, accessed on 6 November 2022), IEEE Xplore (https://ieeexplore.ieee.org/Xplore/home.jsp, accessed on 6 November 2022), and ACM Digital Library (https://dl.acm.org/, accessed on 6 November 2022) databases. The search strings used in this study are composed of the following combination of search terms:

- (“distributed ledger technology” OR blockchain*);
- (“supply chain” OR trackability);
- standard*;
- interoperability.

Currently, there is a significant number of studies in which blockchain technology has been proposed to improve different logistics platforms. This includes the aviation sector, diamond procurement and marketing, and pharmacy supply chains [13,14,15].

As the stakeholders involved in a supply chain have different interests and cannot automatically trust each other, a great amount of time is required to verify the data provided by the suppliers to the customers. To make the corresponding payments, customers must ensure that all accessible data are in order and have not been tampered with by the provider, which slows down the transaction procedure. By maintaining a distributed ledger, stakeholders in a supply chain are always aware of the stored data, preventing unauthorized alterations. Also, the use of blockchain technology allows one to develop smart contracts that launch automatic and almost instantaneous payments, once compliant with the conditions of the contract has been verified.

While there are many benefits to using blockchain technology in logistics systems, there are some problems that the technology itself faces. These challenges must be taken into account so that the most suitable type of technology is chosen for the platform to be developed [16]. One of these challenges is scalability; storing all the generated information, duplicated in all the nodes of a network, gives resilience to access, avoids data corruption, and increases the amount of space needed to store such data. For this reason, it is sometimes not possible to store all the data generated by a platform’s sensors in the blockchain network. Instead, the information is stored outside the blockchain network, i.e., off-chain. Meanwhile, proof that the information has not been tampered with is stored on-chain, as well as the data hashes instead of the full data content [17].

The use of standards in the development of platforms is the key to advancing current technology. The practices that have given good results in previous works are reused in new projects, becoming standards. The use of these practices in new studies makes interoperability viable, as different platforms use the same standardized practices. In the case of supply chains, the literature provides information on a range of standards, from product labeling to device-to-device communications.

An example of standardized logistic labeling is the GS1 Serial Shipping Container Code (SSCC). The GS1 standard is part of a project whose aim is to identify logistic units and harmonize labels across Europe [18]. Other examples used in the literature are the ISO 9001:2015 rules for the management of the quality of products transferred within supply chains [19].

Virtualized assets, exchanged between platforms, should be uniquely identified and their characteristics clearly described. In cases where the transferred asset is fungible, the same features have to be maintained on both systems [20]. By using standards to codify the smart contracts that govern different blockchain platforms, it is possible to keep the characteristics of any asset when transferring it between systems. Different logistics environments that use Ethereum-based networks have used standards such as the ERC721 token interface to represent non-fungible assets and the ERC20, to represent fungible ones [21]. However, this is not enough if each blockchain labels different asset features.

Another way of achieving interoperability between logistics systems is by standardizing the asset labeling. The use of standards makes interaction possible for smart RFID tags and the tracking devices of different providers, enabling the traceability of the system’s products. The authors of [22] made use of GS1 SSCC type of standardized labeling to identify items in the process of developing their Proof of Concept (PoC). Other works that utilized the GS1 standard in the blockchain to identify products, via a unique Global Trade Item Number (GTIN), and facilities, via a unique Global Location Number, are [23,24,25,26,27].

On the contrary, along with the Ethereum token standards, in [28] the authors used the ISO 9001:2015 standard to create globally valid identifiers that are enforced by utilizing bar codes or two-dimensional symbols such as QR codes or alternative RFID tags for projecting physical goods onto digital system standards. In the research of [22], communications between devices inside the trucks of the supply chain utilized the UN EDIFACT standard, a type of standardized format for messages to notify the changes related to products during transportation.

In view of the ethical and scalability issues experienced by most blockchain solutions on the market [29], proposed a standard for the types of data that can be stored on-chain, according to the amount of a certain type of data and the privacy required.

An intermediate mechanism or gateway is needed to enable interoperability between blockchain systems. A gateway makes use of different plugins that exchange information between systems to enable reciprocal operation. As the number of bridged systems increases, so does the number of plugins being utilized. For example, in [30], an agnostic framework was used to act as middleware and addressed interoperability issues between blockchains through the use of the OPENAPI open standard. The standard enabled interaction between different blockchains despite the different methods that they had to store and sign the data they operate. This framework only uses the OPENAPI standard as a means of achieving interoperability between platforms that use it as a gateway to store their information in a blockchain that is more suitable for the generated data. On the other hand, it does not allow for interoperability between platforms that are already deployed and manage their own permissioned blockchains with their own standards.

Since most blockchain developments in the logistics sector make use of permissioned solutions, of which only a set of individuals can be part, interoperability between the different solutions becomes necessary. From the literature review carried out in this section (see Table 1), it is clear that standards have been used in some studies as part of proposals, which does not imply interoperability between platforms that use different standards. Interoperability can only be achieved if the same standards are used. However, to the best of the authors’ knowledge, no research has addressed the challenge of interoperability among systems based on different blockchain solutions. We have proposed a novel model, added as a gateway to the existing platforms, that allows for the implementation of a system, which enables the verification of information, regardless of how it has been encoded.

## 3. Proposed Model

The proposed model consists of two correlated parts: the data structure and the protocol process, both described in this section. A data structure defines how an asset is stored within the supply chain as a verifiable credential, aligned with the W3C Verifiable Credential Data Model standard for expressing verifiable credentials on the Web. The protocol process describes procedures on the verifiable credential in the context of tracking assets in the blockchain-based supply chain. In Section 3.1 that follows, we explain how a verifiable credential works, as the basis of the data model presented in Section 3.2. Finally, the protocol that the proposed model must follow is described in Section 3.3.

### 3.1. Verifiable Credentials

In the physical world, credentials are used continuously. An example of such a credential is a driver’s license, which verifies that the holder of the licence is capable of operating a motor vehicle. Another, even more illustrative example is that of a government-issued passport, which proves a person’s identity when travelling abroad. The usage of these credentials in the physical world is typically of some benefit to us. However, the use of such third-party verified credentials in digital form on the Web is still difficult, and it is challenging to obtain the same benefits from using digital credentials as from physical credentials in the physical world [31].

The standard related to expressing verifiable information on the Web has been published to provide a regulation specification for expressing credentials on the internet, so they are machine-verifiable, respect privacy, and, most importantly, are cryptographically secure. According to this standard, a verifiable credential is defined as a tamper-evident credential with authorship, which can be cryptographically verified [31]. A basic ecosystem of verifiable credentials consists of the entities called the holder, the issuer, the subject, the verifier, and the verifiable data registry, along with the relations established between all of them.

The holder, the issuer, the subject, and the verifier are entities with distinct and independent existence (e.g., persons, organizations, or devices) within which the ecosystem performs one or more roles. The issuer asserts claims about some subject, creating a verifiable credential from these claims and then issuing and delivering the verifiable credential to the corresponding holder. The subject is an entity about which claims are made, and in many cases, it is directly related to a verifiable credential holder. The holder stores one or more verifiable credentials received from the issuer in their credential repository (e.g., vault or personal verifiable credential wallet).

Furthermore, the holder of the credentials has the autonomy to decide about presenting their verifiable credentials to other entities, i.e., verifiers. Typically, the verifier requests the holder to present verifiable credentials issued by the issuer. In the case that the holder decides to present these credentials, they can be later cryptographically verified by the verifier.

The verifiable data registry mediates in the creation and verification of data, an important role in the verifiable credential ecosystem required to verify the user credentials. Verifiable registries can be implemented as trusted databases, decentralized databases, government ID databases, or distributed ledgers (e.g., blockchain). Often, within the usage of verifiable credentials, more than one type of verifiable data registry is utilized.

**Figure 1:**
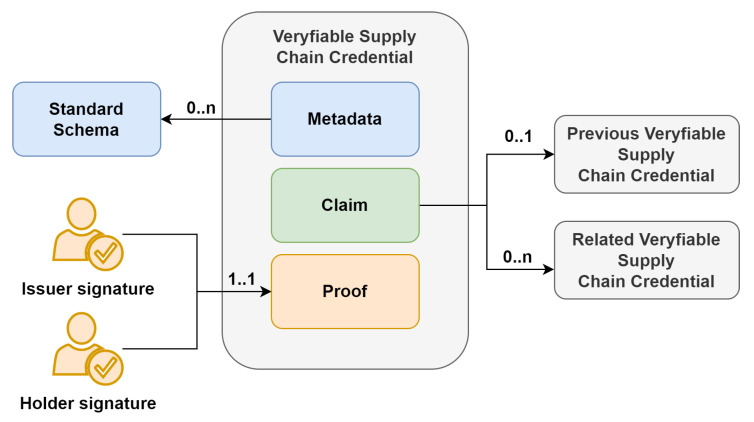
Structure and data model of the Verifiable Supply Chain Credential.

The main functions in verifiable credentials are implemented by utilizing public-key cryptography, mainly through a digital signature mechanism that enables that verifiable credentials to be more tamper-evident and trustworthy than physical ones. A verifiable credential, see Figure 1 for reference, is a set of one or more claims asserted by the same issuer and expresses some statements about the subject, that can be cryptographically verified at any time. These claims are stored in the dedicated verifiable credential claim component.

Next to the claim component, the fundamental components of verifiable credentials are also the metadata and proof components. Pieces of information as a credential identifier, holder identifier, issuer identifier, expiration, verification services, and other pieces of information that are part of the credential metadata component. The most critical data stored in this component is the verifiable credential schema. This schema, also named as Context, is typically, but not always, specified in the form of the JSON-LD file [32]. A schema defines a data model, i.e., attributes (e.g., holder, issuer, etc.), and their formats, used in the verifiable credential claim and proof components.

Additionally, an important feature of the verifiable credentials is the ability of the data model to be extended. One schema’s data can be extended with other new machine-readable schemas specified in a verifiable credential metadata component. As a consequence, one verifiable credential can include multiple schemas, extended from the initial W3C Verifiable Credential Data Model [31], with newly defined attributes. To ensure that verifiable credentials with multiple defined schemas in the metadata component align with the standard for expressing verifiable credentials on the Web, the initial data model, i.e., W3C Verifiable Credential [31], must always be defined in the verifiable credential metadata component. If the verifiable credential data model is extended, the verifiable credentials’ semantic interoperability is guaranteed because of the mechanisms and procedures mentioned above.

A verifiable credential indeed becomes verifiable when the set of tamper-evident claims, collected in the claim component and credential metadata stored in the metadata component, are joined together in one piece and are later digitally signed by the issuer. The results of such an operation, i.e., digital signatures, are collected in a verifiable credential proof component, which enables anyone, to whom the verifiable credential is presented, to cryptographically prove who is the issuer of verifiable credential claims. Proof of verifiable credentials can be created using different types of cryptographic mechanisms for digital signing. Furthermore, the utilization of digital signature provides integrity to the verifiable credentials—a warranty that the verifiable credential was not altered during transmission and is presented with the data, i.e., claims, in the same form as was created by the issuer. Moreover, an issuer cannot deny the authorship of claims related to the subject of verifiable credentials after the verifiable credential has been issued.

The proofs are required to establish trust inside the verifiable credential ecosystem, namely, to establish trust between the issuer and the verifier, where the verifier trusts the issuer to issue the credentials with data that are relevant to them. All entities must trust the verifiable data registry; because it is tamper-proof, the data it stores are correct. The verifier and the holder must trust the issuer that they assert valid claims about the subject. The holder needs to trust that the repository, where their verifiable credentials are stored, is secure. This trust model is distinct from other models because it ensures that the verifier and the issuer do not need to trust the holder’s verifiable credentials repository, i.e., a third party. However, they can directly interact with each other and trust only in successfully, cryptographically verified credentials. Furthermore, the issuer does not need to know nor trust the verifier, but they can assert claims about the holder themselves.

### 3.2. Data Model

In the proposed model, we extend the verifiable credential outlined in Section 3.1 for asset tracking (e.g., pharmaceutical products) with blockchain-based supply chains. Such a model enables interoperability in terms of all the alterations related to the tracked assets, registered in various blockchain-based supply chain systems. These alterations can be cryptographically verified. At the core of the model is a dedicated verifiable credential named the Verifiable Supply Chain Credential (VSCC), depicted in Figure 1.

We extended the W3C Verifiable Credential Data Model 1.1 (W3C-VC-schema) schema (https://www.w3.org/TR/vc-data-model/, accessed on 10 January 2023) with two additional schemas, namely “VSCC-schema”, and “Asset-Standard-schema”. As can be observed in Figure 1, these two schemas, marked in blue and green, are specified in the VSCC metadata component inside the “@context” attribute, beside the initial W3C-VC-schema.

The VSCC-schema defines a dedicated data model, including a set of attributes related to the logistics field. These attributes are later utilized in the claim and proof component of VSCC. The central, but not the only, attribute defined in the VSCC-schema is the “blockchainSupplyChainData”. This is utilized in the claim component and includes the following in the supply chain tracking related attributes: “id,” “assetData,” “previousCredential,” “ relatedCredentials,” and “verificationService” (in Figure 1, marked in blue color). The value of attribute “id” corresponds to a verifiable credential subject identifier, i.e., an asset that is tracked in a blockchain-based supply chain system.

Attribute “assetData” contains complete data on the tracked asset. Furthermore, asset data have been specified with VSCC using the attributes defined in “Asset-Standard-schema”. This schema’s inclusion enables asset data to be written with already established and widely accepted standardized vocabularies, e.g., GS1, EN ISO 9000, etc. Moreover, “Asset-Standard-schema” can be manually chosen, depending on the requirements of the sector to which the tracked asset relates.

The first phase of tracking is implemented with the content of the “previousCredential” attribute. If some previous version of the current tracked asset exists, it is specified in this attribute. In such a case, the whole related VSCC, including metadata, claim, and proof components, are defined in this attribute. In the case that the current asset is the first one in the supply chain, the content of the “previousCredential” attribute is annotated with the value null. The purpose of the “relatedCredentials” attribute is similar, however, unlike the “previousCredential” attribute, in this attribute, a set of assets can be specified (their VSCC data), that are not directly related to the history of the currently tracked asset, but are somehow related to the tracked asset.

It is important to note that content of “previousCredential”, and “relatedCredential” attributes is specified in the form of VSCC. Such design allows the content of these attributes, which describe the historical status of a tracked asset or its other related assets, to be cryptographically verified from each VSCC, as well as from the tracked asset itself.

The second phase of the tracking is implemented through the “verifiactionService” property, which connects tracked asset VSCC with a particular blockchain-based supply-chain system where this VSCC is registered. Herein, the blockchain network type of the supply-chain system is not limited and can be either permissioned or permissionless. The minimum requirements for the verification of the asset represented with VSCC in the blockchain system, are described below.

First, the tracked asset’s information is registered on a blockchain-based system, whereby the minimum pieces of information that must be stored on the blockchain are the VSCC identifier, the tracked asset identifier, and the hash value of VSCC data. Second, these asset data, registered on the blockchain-based supply chain system, must be available to verifiers on-demand. These requirements can be achieved with proper pieces of information of the blockchain-based supply chain system on which the tracked asset is registered, and are specified in VSCC attributes “info”, “endpoint”, and “address” that are part of the “verificationService” property.

The description of the blockchain-based system is specified in the “info” attribute and can include data related to the type of blockchain or a verification service. For example, but not limited to, blockchain type (e.g., permissioned, public-permissioned, permissionless) or service type (e.g., RPC, HTTP, etc.) can be specified within this attribute.

In the “endpoint” attribute, data about the method, and the attributes required for gathering the pieces of information about a tracked asset, must be specified. The address of where the aforementioned data can be obtained is specified in the “address” attribute and can include a smart contract blockchain address, HTTP server address, etc. These three attributes under “verificationServices” property must provide the verifiers with the minimum data required by the blockchain-based system where the tracked asset is registered. This is to enable them to perform a proof-of-existence verification of the selected VSCC. In this case, proof-of-existence verification guarantees that the tracked asset, i.e., the VSCC which is the verification subject, has been registered in an asset-related process in some blockchain-based supply-chain system.

It would be possible to use a pointer as the one referencing blockchain information in the “textitverifiactionService” property. Although that level of optimization is not accounted for in comparison to the double layer of security gained by replicating the VSCC within each new VSCC. Thus, if an attacker has been able to manipulate the information of a historical credential stored in the IPFS, it is possible to retrieve that information from the most recent one, with the historical credential record.

Moreover, the verifiable credential proof component is extended with two attributes, i.e., “issuerProof”, and “holderProof.” Typically, the verifiable credential (e.g., W3C-VC) includes only one proof, i.e., a credential issuer’s digital signature. This may result in the holder, to whom the credential is issued, becoming the credential owner, even though they may not want to. In some cases, as in the case of tracking assets in the supply chain, it may happen that some entity, i.e., an issuer, assigns the ownership of a tracked asset to another entity, i.e., holder, without that holder’s wish to become the new owner of a tracked asset. For these reasons, for the VSCC to be successfully cryptographically verified, the VSCC must contain proofs, i.e., the digital signatures of both entities, i.e., issuer and holder. With such design, the issuer cannot assign the ownership or change the content (i.e., “description”) without that holder’s (new or actual) confirmation of this through their digital signature, i.e., “proof”.

### 3.3. Protocol Model

There are two fundamental processes described below, namely, verification of VSCC and the issue of VSCC, where the first one, i.e., verification, is the essential component of the second, i.e., issuing process.

#### 3.3.1. Verify Verifiable Supply Chain Credential

As already described, VSCC consists of three fundamental components, i.e., metadata, claim, and proof. The metadata component includes data related to the credential itself. The claim component combines a specification of the tracked asset and proof-of-existence blockchain verification services information. Furthermore, the proof component includes digital signatures from all entities involved in operations related to a tracked asset.

For successful verification, two conditions must be met. First, the digital signatures of all entities from the proof component must be cryptographically verified. Second, the proof-of-existence data, i.e., a hash of VSCC, acquired from verification service, must be equal to the hash of VSCC data presented to a verifier. Algorithm 1 shows the pseudocode of the verification procedure. The verification can only be successful if all the VSCCs that are part of the VSCC, i.e., previous and related credentials, are also successfully verified.

There may be two reasons for failure in the verification of the VSCC. First, the digital signatures of the presented VSCC are invalid, e.g., the VSCC has been altered and does not present the data signed by entities, i.e., the holder or the issuer. Second, the VSCC presented to the verifier is not the VSCC registered in the blockchain-based supply chain system, i.e., a hash of VSCC is not equal to the hash acquired from the verification service specified in the presented VSCC.

#### 3.3.2. Issue Verifiable Supply Chain Credential

**Figure 2:**
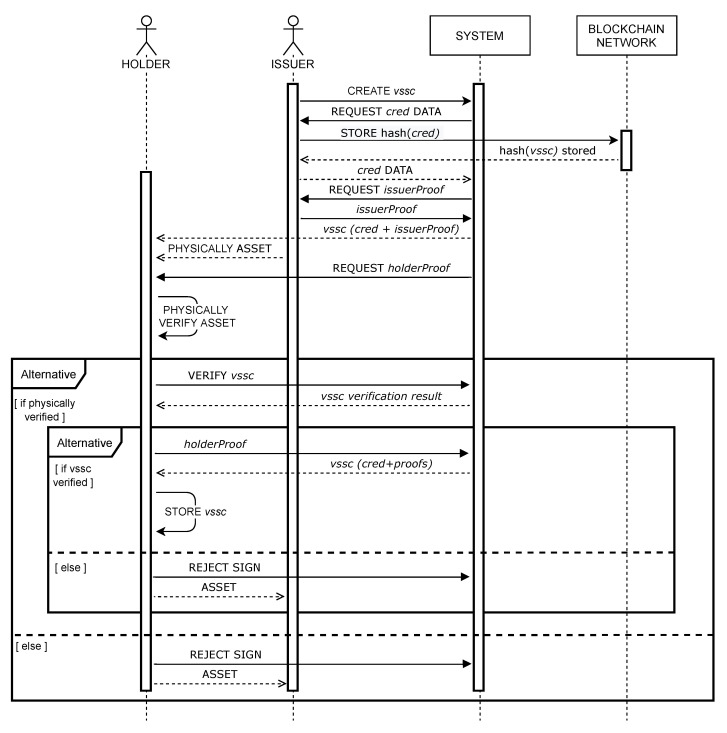
Verifiable supply chain credential issuing procedure.

Figure 2 shows the sequence diagram related to the procedure of issuing the VSCC. The issuance of the VSCC is required if a new asset is produced and is to be tracked with a blockchain-based supply chain system or if some specification (e.g., country, owner, etc.) of an existing tracked asset has been changed.

The process begins with the issuer, who needs to issue a VSCC. An issuer, who is also the current owner of an asset, is requested to deliver data that describes the tracked asset, i.e., pieces of information specified in VSCC metadata and claim components. After the data describing the asset is prepared, the draft of VSCC is prepared, including all the data related to metadata and claim components. The data of these two components are then merged, and denoted as “cred” in Figure 2.
**Algorithm 1: **Pseudocode of the *verify(vscc)* method for verifying a Verifiable Supply Chain Credential
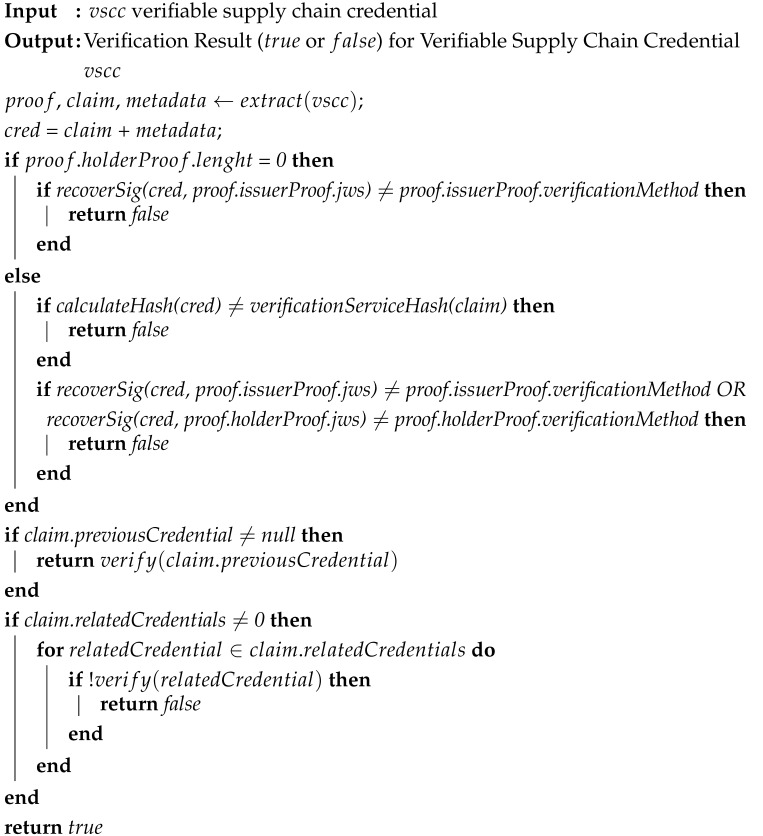


Before the issuer forwards the previous data (i.e., “*creed*”) back to the system, its hash is generated and registered in the blockchain network. Then, the issuer is requested to digitally sign these data, and as a result, produce the issuer proof. The VSCC now consists of all three components, where the proof component only includes the issuer proof.

Next, the VSCC is presented to the holder, who also acts as a verifier in the issue procedure. At the same time, the physical asset is also presented to the holder, who physically checks that the physical asset is indeed described with the presented VSCC. In the case that a physical check of asset fails, the holder can decline the issuance procedure. The same can occur if the cryptographic verification of the VSCC performed with Algorithm 1 fails.

If, on the contract, the verification is successful, the holder digitally signs the data of the verified VSCC and produces holder “*proof*”, which is added to the final VSCC. The final VSCC remains stored within the holder agent. As a result of the issuance procedure, VSCC includes both proofs, i.e., from the issuer and the holder. The data in the VSCC describe an asset, and all the operations related to tracked assets are cryptographically provable.

## 4. Use Case Scenario: Pharmaceutical Supply Chain

To evaluate the proposed model, its application has been simulated. The pharmacy sector has been chosen as the case scenario, specifically, an aspirin manufacturing supply chain, using different blockchain technologies at each of the stages of the process. The process consists of obtaining and transforming chemicals, such as phenol and sodium carbonate, into acetylsalicylic acid, commonly known as aspirin, apt for human consumption. The steps followed in the described scenario are production, packaging, distribution, and retail sale. For each stage, a certificate is needed that guarantees the quality of the pharmaceutical product, especially at the production stage, where the product needs four chemical reactions, each having its own parameters [33]. The reactions consist of five steps: (i) the storage of the chemicals, (ii) preparation, (iii) reaction, (iv) purification, and (v) the storage of the product.

**Figure 3:**
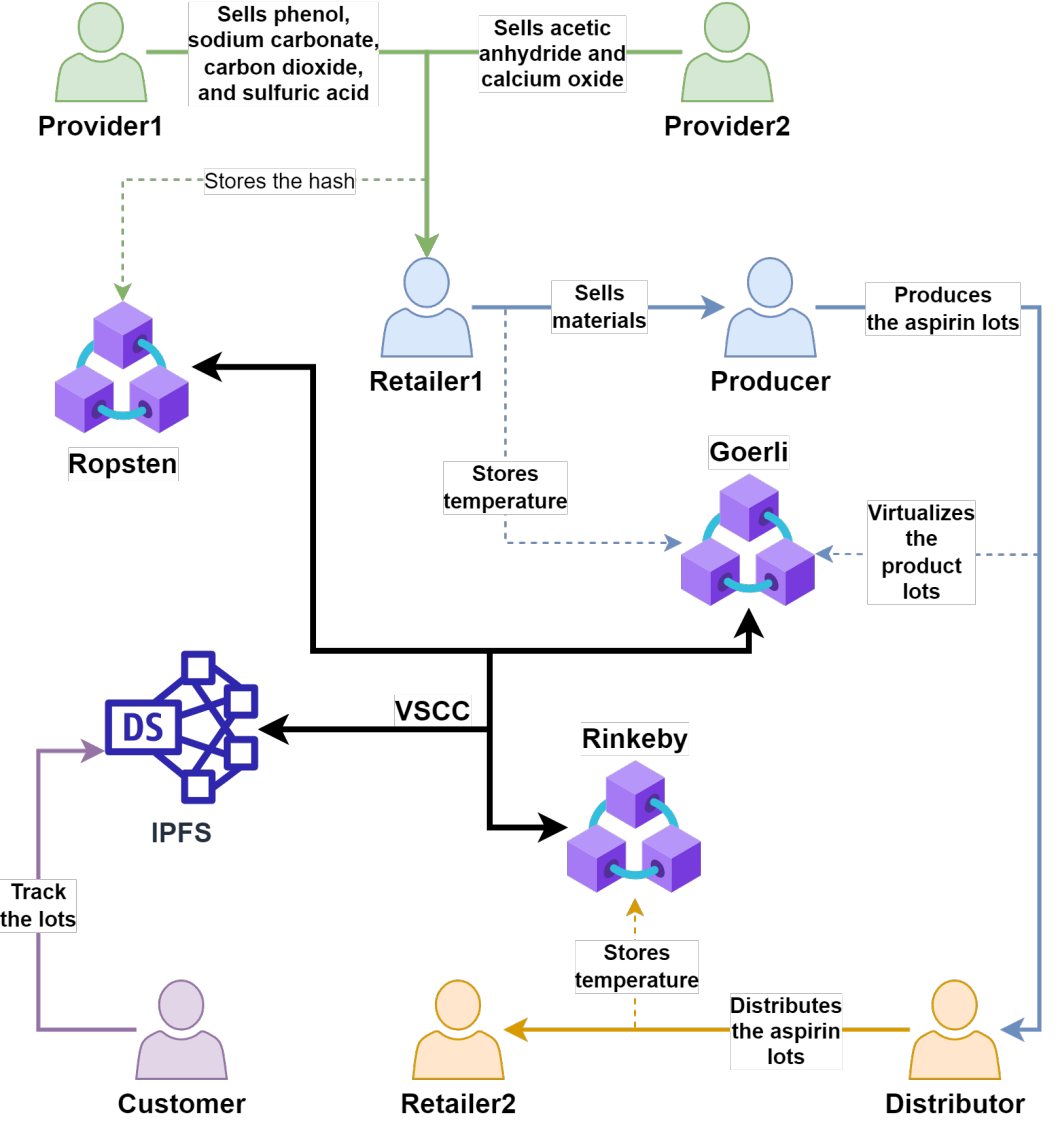
Diagram of the simulation carried out in a pharmaceutical blockchain-based supply chain scenario. It is shown how the different stakeholders are grouped together when they are part of the same platform (exemplified as the usage of different blockchain networks during the simulation). In the diagram it is possible to see how each stakeholder interacts with its own blockchain and how each blockchain is related through the VSCCs to the IPFS. Finally, the relationships between the stakeholders and the consumer during the tracking process are shown.

In a typical pharmacy blockchain-based supply chain [15], the pharmaceutical asset is constantly monitored, storing those data in a blockchain as a log. The proposed framework makes those data universally accessible by any stakeholder that interacts with the asset, without forcing them all to use the same blockchain solution. In our experiment, see Figure 3, an aspirin lot is presented in the form of a Verifiable Supply Chain Credential. At each stage, a record of the tracked lot is registered in the blockchain network. Additionally, each entity, except the Consumer, holds a digital identity with the appropriate private key. The proof component includes the digital signatures of the relevant entities—depending on the stage. For the experiment, we have outlined the following scenario with the following entities that satisfy the above-mentioned case study:

1. In the first stage, the Producer, who is responsible for the process of creating the aspirin lot, is in Spain. The process consists of four reactions. For the purpose of simplicity, the stages of a reaction are avoided in the simulation, and the focus is placed on two parameters for each reaction: temperature and pressure. These parameters are stored in a blockchain, to have a tamper proof historic of the aspirin lot.
2. The ones that provide the chemicals needed to make the reactions are Provider1 and Provider2. From Provider1, the Producer buys phenol, sodium carbonate, carbon dioxide, and sulfuric acid. While Provider2 is the one who sells acetic anhydride and calcium oxide. All those assets are sold to the Producer by a retailer, Retailer1, in Portugal. For this purpose, the temperature to which the chemicals are exposed during their storage stage is stored in the blockchain.
3. In the packing stage, the Producer mills the acetylsalicylic acid obtained from the last reaction and packs it in pills, creating boxes with aspirin lots. Here, properties of the boxes, such as the size and number of pills stored, as well as the provenance, are stored in the blockchain.
4. The Distributor delivers the final product to retailers in different cities. Storing blockchain properties, such as the temperature at which the lot has been exposed.
5. Retailer2 is a retailer from Slovenia who received the aspirin lot from the Distributor.
6. The customer can verify the pharmacy supply chain—from the chemicals used and the reactions carried out, to the packaging of the aspirin box, which is sold by the Retailer.

Furthermore, a real-world situation has been considered where some entities are part of the same blockchain network (e.g., consortium) for blockchain-based supply chain systems. In our scenario, stakeholders have their own blockchain-based platforms, and are grouped into consortiums as follows: Provider1 and Provider2; Producer and Retailer1; and Distribution and Retailer2. The simulation has been carried out to validate the proposed model, where entities use separate blockchain-based systems that are unrelated to each other for asset tracking.

To allow for the validation of the proposed model, three different publicly available test blockchain networks (Ropsten, Rinkeby, and Goerli) have been used, where a total of three prototypes of blockchain-based supply chain systems are deployed. Each blockchain-based supply chain system is implemented with a smart contract representing a minimal viable blockchain component of the supply chain system whose basic task is to store the unique identifier of VSCC, the unique identifier of a tracked asset, and a hash value of VSCC. It is important to note that utilized blockchain networks have different characteristics, and despite being publicly open, they can be considered as different types of blockchain networks (e.g., public-permissioned, permissioned, consortium, private, etc.).

### 4.1. Analysis

As mentioned previously, within the proposed model, pharmaceutical assets are presented by VSCCs in the form of JSON files. All the VSCCs that have been generated during the experiment are available on a public GitHub repository (https://github.com/YerayMM/VSCCs, accessed on 10 January 2023), while each directory signifies the internal credential storage of the entity.

Each entity in the simulated use case owns a digital identity, used for generating VSCC proofs. Furthermore, each entity incorporates a blockchain network where the hash value (i.e., proof-of-existence) of the VSCC is registered. The aforementioned information is specified in Table A1 in Appendix A. The results of the simulation of our model in the use case scenario are shown below:

1. Provider1 produces carbon dioxide, phenol, sodium carbonate, and sulfuric acid and issues a VSCC (carbon-dioxide-provider1-vscc.json, phenol-provider1-vscc.json, sodium-carbonate-provider1-vscc.json, sulfuric-acid-provider1-vscc.json) as a digital representation of each physical asset. The tracked asset attributes, i.e.,: name (Phenol) and country (Spain) are defined with the GS1 standard. A hash value of each VSCC is registered on the Ropsten blockchain network (within the smart contract address 0xeD644b4B7842f260707417CF1b163a8fF6deeA27). The issued VSCC contains two proofs, i.e., issuerProof, and holderProof. Both proofs, i.e., digital signatures, are generated by Provider1 because the asset was produced by them, and the production of assets is an event that is tracked with a blockchain-based supply chain system. For the same reason, the issuer (i.e., previous asset owner), and the holder (i.e., current asset owner) have the same digital identity address (i.e., from the Provider). Provider2 produces acetic anhydride and calcium oxide (acetic-anhydride-provider2-vscc.json, calcium-oxide-provider2-vscc.json) and follows the same logic as Provider1.
2. Retailer1 imports the chemicals from Provider1 and Provider2, and as a result of this event, a new VSCC is issued for each imported asset (acetic-anhydride-retailer1-vscc.json, calcium-oxide-retailer1-vscc, carbon-dioxide-retailer1-vscc, phenol-retailer1-vscc, sodium-carbonate-retailer1-vscc, sulfuric-acid-retailer1-vscc). The tracked asset attributes, i.e.: name (Phenol) and country (Spain) are defined with the GS1 standard. A hash of VSCC is registered on the Goerli blockchain network (within the smart contract in address 0x1b6626b19f3AA7867BDb849E57c3C21c78238B96). The issued VSCC in the “previousCredential” attribute includes a previously issued VSCC (i.e., phenol-provider1-vscc). Proofs in issued VSCC contain issuerProof—a digital signature generated by the previous owner (issuer) of a tracked asset, i.e., Provider1, and holderProof—a digital signature generated by the current owner (holder) of a tracked asset, i.e., Retailer1.
3. The producer imports the chemicals from Retailer1, following the same logic as described above. With the purchased chemicals, the Producer carries out four reactions, producing: (i) sodium phenolate (sodium-phenolate-producer-vscc.json) from phenol and sodium carbonate; (ii) sodium salicylate (sodium-salicylate-producer-vscc.json) from the sodium phenolate and carbon dioxide; (iii) salicylic acid (salicylic-acid-producer-vscc.json) from the sodium salicylate and sulfuric acid; (iv) acetylsalicylic acid (acetylsalicylic-acid-producer-vscc.json) from the salicylic acid, acetic anhydride, and calcium oxide. Each reaction has its VSCC issued, storing additionally temperature and pressure, i.e., name (Acetiylsalicylic acid), country (Spain), temperature (40 ºC), and pressure (1 atm); all the attributes are defined with the GS1 standard. A hash of VSCC is registered on the Goerli blockchain network (within the smart contract in address 0x1b6626b19f3AA7867BDb849E57c3C21c78238B96). The issued VSCC in the “relatedCredential” attribute includes data about the chemicals used, i.e., VSCC (phenol-retailer1-vscc.json). The newly created VSCC contains two proofs, i.e., issuerProof, and holderProof. Both proofs, i.e., digital signatures, are generated from Producer1 because that asset was produced by them, and for the same reason, as detailed for the providers, the issuer (i.e., previous asset owner), and the holder (i.e., current asset owner) have the same digital identity address (i.e., Producer). Finally, another VSCC (aspirin-packing-producer-vscc.json) is issued from the Producer, after milling the acetiylsalicylic acid, obtained from the last reaction, and creating the boxes for the aspirins. The weight of each aspirin is stored and follows the same logic as the one detailed for the chemical reactions.
4. The Producer transfers aspirin lots via Distribution to a new location, and the VSCC (aspirin-distribution-vscc.json) that is issued, reflects this event. The tracked asset, i.e., name (Aspirin) and country (Spain), is defined with the GS1 standard. A hash value of VSCC is registered on the Rinkeby blockchain network (within the smart contract address 0xf409f74a81006E031085218D6c83b71443cE18aa). The VSCC issued in the “previousCredential” attribute includes data from the VSCC (tomato-sauce-processor-vscc.json), which is a subject of distribution. Proofs in the issued VSCC follow the same logic as the proofs described previously in the transference of assets: issuerProof, i.e., Producer, and holderProof i.e., Distribution.
5. Retailer2, in Slovenia, receives the aspirin lot from Distribution, and as a result of this event, a new VSCC (aspirin-retailer2-vscc.json) is issued. The tracked asset, i.e., name (Aspirin) and country (Slovenia), is defined with the ISO standard. A hash value of VSCC is registered on the Rinkeby blockchain network (within the smart contract address 0xf409f74a81006E031085218D6c83b71443cE18aa). The VSCC issued in the “previousCredential” attribute includes the previously issued VSCC for the aspirin lot (aspirin-distribution-vscc.json). Proofs in the newly issued VSCC contain issuerProof—a digital signature generated by the previous owner (issuer) of a tracked asset, i.e., Distribution, and holderProof—a digital signature generated by the current owner (holder) of a tracked asset, i.e., Retailer2.
6. The final VSCC (aspirin-retailer2-vscc.json) digital presentation of the aspirin lot is available to the Customer for verification and consists of all VSCCs that are part of the pharmacy supply chain—from chemicals and reactions to the aspirin box. With Algorithm 1 the Customer can now verify this VSCC. First, the hash value of the presented VSCC (aspirin-retailer2-vscc.json) is calculated, and compared with a hash value registered on the blockchain network (information about blockchain network is acquired from VSCC). Next, the proofs, i.e., digital signatures of the VSCC (aspirin-retailer2-vscc.json) from Retailer2 and Distribution, are verified. After that, all the VSCCs that are included in “previousCredential”, and “relatedCredential” attributes are verified in the same manner, i.e., comparison of VSCC hash and digital signature validation.

In this section, a typical use case scenario has been described, in which several actors carry out the process of buying, selling, and transforming resources and materials to create a final product (aspirin). Thanks to the model proposed in this work, the end-user, who bought the product from the retailer, can trace back the final product even if each intermediary stores their related information in a different blockchain network, demonstrating the interoperability achieved thanks to the proposed model.

Negotiations between agents within a logistic platform belong to a logical layer that underpins the proposed model. Since each negotiation is carried out independently between the peers of the platform, the addition of the proposed model does not affect the performance of a platform with a great number of agents. Moreover, each product can only have one credential in force, the last one, which encompasses all the others. These credentials are a JSON file in which each stage of the product in the logistics system, is recorded. No matter how many stages there are, a credential does not grow to sizes larger than megabytes in the worst case. This also does not affect the performance of any blockchain-based logistics system, as IPFS is the system in charge of managing the storage of the credentials, while the blockchain network is used to store the hash of the file that verifies the credential’s credibility.

## 5. Discussion

The proposed model has been applied to a simulated real-world scenario, as described in Section 4, the purpose of which was to answer two research questions and confirm four hypotheses. The aim was to verify whether the proposed model provided interoperability among different blockchain-based supply chain systems and whether the tracked assets could be defined in different standardized formats. The results of the analysis confirmed all four hypotheses raised in Section 4.1:

- With the proposed model, the asset can be tracked among different blockchain-based supply-chain systems. From the analysis, it can be observed that the asset was successfully tracked among six different blockchain-based supply chain systems, built on top of four blockchain networks with different characteristics. Because of this, we can say that Hypothesis 1 was confirmed.
- With the proposed model, the tracked asset’s supply chain can be cryptographically verified, even though the asset was tracked among different blockchain-based supply-chain systems. From the analysis, it can be observed that the supply chain asset was successfully verified with validation of the proofs, i.e., digital signatures that are part of VSCC that represent a tracked asset, and a comparison of the VSCC hash value with the hash value registered on the blockchain network. Because of this, we can say that Hypothesis 2 was confirmed.
- With the proposed model, a tracked asset can be defined in a standardized format. The analysis shows that the tracked asset was defined with GS1 and ISO 22005-2007 standards at different stages of the supply chain. Because of this, we can say that Hypothesis 3 was confirmed.
- With the proposed model, a tracked asset can be described with several different standardized formats. The analysis shows that the same tracked asset was defined with GS1 and ISO 22005-2007 standard at different stages of the supply chain. Because of this, we can say that Hypothesis 4 was confirmed.

To conclude the discussion, it can be said that the proposed model ensures interoperability between the systems that implement it. Additionally, regarding the existing literature, studied in Section 2, the proposed model (i) provides flexibility in terms of the use of standards and blockchain platforms, (ii) optimizes the storage of on-chain data so it is no longer needed to duplicate the information shared between blockchain platforms, and (iii) provides a gateway that facilitates the implementation of tracking applications for the end consumer, while it does not affect the scalability of the logistic platforms that implement it. On the other hand, the model implements an extra layer of complexity to the existing platforms, which is either way necessary if interoperability between them is to be achieved. Finally, another drawback of the model is the General Data Protection Regulation (GDPR) [34] where the storage of public information in the distributed storage system is regulated, as are the public keys of the actors that interact in the supply chain platforms. Finally, we could say that the most difficult part of the design of such a model was to find a way to allow the verification of the transactions and transformations of the assets carried out during each of the supply chains, while extrapolating to other kinds of use cases, and maintaining the flexibility and independence of each platform to take its own decisions.

## 6. Conclusions

In this article, a novel model has been designed to tackle the interoperability challenges among blockchain-based supply chain systems. The difficulties related to virtualizing physical data on a tracked asset with different standardized formats have also been addressed. To address the problem of interoperability between different blockchain-based supply chains, the use of verifiable credentials has been proposed. These credentials follow the recommendations issued by the World Wide Web Consortium and enable the expression of credentials secured by cryptography on the web.

The proposed model uses verifiable credentials for the traceability of assets between different blockchain-based supply chain systems. For this purpose, a data model has been developed to issue verifiable credentials about assets whose traceability information may have been stored in different blockchain-based supply chains. The designed model was evaluated through a logistics sample scenario in which a series of entities intervene, each with a well-defined role (i.e., Provider, Producer, Distribution, Retailer, Consumer). This scenario has shown how the end consumer can trace the route of an aspirin batch from the origin (i.e., chemicals and reactions carried out).

This work has proposed a series of hypotheses (Hypotheses 1–4) that, thanks to the use of the proposed model, have been verified: (1) interoperability between different blockchain systems; (2) the virtualized asset is cryptographically verifiable; (3) the asset can be digitized in a standard format; and (4) the asset can make use of different standard formats during the life cycle of the logistics system.

This work demonstrates that the proposed model provides interoperability between different supply chains based on blockchain technology, allowing the virtualized object to be cryptographically verifiable. Additionally, tracked assets can be defined with different standardized formats during their supply chain lifecycle. Since the proposed model focuses on expressing tracked assets in digital form that can be used among different blockchain-based supply chain systems, other features (e.g., digital identities, credential presentations, etc.) are beyond this article’s scope. In the future, the proposed model will be implemented in an industrial environment and its operation will be demonstrated.

Moreover, it has been possible to verify that the model is scalable in logistics systems. The number of agents involved in the process does not affect the performance of the model, as they do not deal with the negotiations between them, but only with the creation of a chain of verified credentials that allow for the traceability of the products. Since the credentials are stored in JSON format, and only have text fields, the size of the credentials, including the stages through which the tracked asset has passed and its other components (with their respective stages and information), is acceptable in a realistic scenario. The blockchain technology used by each agent is only necessary for the storage of the credential hash, with the sole purpose of providing confidence that the credential has not been modified after its creation.

On the basis of the research presented in this paper, it can be concluded that the use of the proposed model in blockchain-based logistics platforms provides a substantial improvement (interoperability, verifiable traceability, use of standards, and interoperability between different standards), enabling its use without negatively affecting the performance of such platforms. Regarding future lines of research, it will be necessary to find a way to mask the information of the actors that interact in the supply chains so it is possible to comply with the GDPR.

## Figures and Tables

**Table 1:** Comparative survey of the relevant literature that had been identified.

Title	Summary	Means of Achieving Interoperability
Blockchain interoperable digital objects [20]	Theoretical work that clarifies the categorization of different crypto assets.	Definition of standard attributes. Not a real solution to the interoperability problem between platforms that have already been deployed.
Tracing manufacturing processes using blockchain-based token composition [21]	Framework that allows the traceability of assets.	Use of coding standards as a way to operate between platforms. Not a solution to the problem of interoperability between different blockchain-based platforms.
Real-time supply chain—A blockchain architecture for project deliveries [22]	Tracking framework that makes use of RFID and GS1 standards.	Use of standards in the virtualization of the assets. Does not allow interoperability between platforms that use other kind of standards.
Blockchain-based supply chain traceability: Token recipes model manufacturing processes [28]	Blockchain-based traceability platform that makes use of the ISO 9001:2015 standard.	Use of standards in the virtualization of the assets. Not allowing interoperability between platforms that use other kinds of standards.
Blockchain interoperable digital objects [29]	Industrial blockchain-based framework for the sharing of data between platforms.	Definition of standard attributes. Not a real solution to the interoperability problem between platforms that have already been deployed.
Toward a policy-based blockchain agnostic framework [30]	Framework that acts as a gateway to achieve interoperability between blockchain-based platforms.	The achieved interoperability is related to the use of different blockchain networks depending on the context of the use case. It is not used as a means of enabling interoperability between platforms that have already been deployed and are functional. It does not allow interoperability between different platforms that use different standards to store the data.

## Data Availability

Data used to conduct this study can be found in https://github.com/YerayMM/VSCCs (accessed on 20 December 2022).

## Appendix A

**Table A1:** Entities digital identity and blockchain supply-chain system addresses.

Entity	Digital Identity Address	Blockchain Name and Address
Provider1	0xd671ede1de69f77abf778dad82f2a07bdb136790	Ropsten—0xeD644b4B7842f260707417CF1b163a8fF6deeA27
Provider2	0x1bb7d2dc077d270a0bebc9d5606585dfb88b4fda	Ropsten—0xeD644b4B7842f260707417CF1b163a8fF6deeA27
Retailer1	0x98577132b8b98a7c5da0ae49272399cdb19b2adc	Goerli—0x1b6626b19f3AA7867BDb849E57c3C21c78238B96
Producer	0xcbbea4d3fea9d5395509445ee45eee0da7b4e166	Goerli—0x1b6626b19f3AA7867BDb849E57c3C21c78238B96
Distribution	0x802bc8babf61a4a706ca8abd2302d23921ab3783	Rinkeby—0xf409f74a81006E031085218D6c83b71443cE18aa
Retailer2	0x478a3211d43cca0e30075f89b972043df83114c4	Rinkeby—0xf409f74a81006E031085218D6c83b71443cE18aa
